# Supplementary material for: Chemo-mechanical diffusion waves explain collective dynamics of immune cell podosomes
Source: Nat Commun. 2023 May 22;14:2902. doi: 10.1038/s41467-023-38598-z (PMC10202956; doi:10.1038/s41467-023-38598-z)
Supplement: Supplementary file 1 — Supplementary information [file 41467_2023_38598_MOESM1_ESM.pdf]

Supplementary Information
for
"Chemo-mechanical Diffusion Waves Explain Collective Dynamics of Immune Cell Podosomes"

Ze Gong[1,2], Koen van den Dries[3], Rodrigo A. Migueles-Ramírez[4,5,6], Paul W. Wiseman[4], Alessandra Cambi[3], Vivek B. Shenoy[1,7,]*

[1]Center for Engineering Mechanobiology, University of Pennsylvania, Philadelphia, PA, USA 19104;

[2] CAS Key Laboratory of Mechanical Behavior and Design of Materials, Department of Modern Mechanics, University of Science and Technology of China, Hefei, Anhui 230027, China

[3]Department of Cell Biology, Radboud Institute for Molecular Life Sciences, Radboud University Medical Center, Nijmegen, Netherlands;

[4]Departments of Chemistry and Physics, McGill University, Otto Maass (OM) Chemistry Building, 801 Sherbrooke Street West, Montreal, QC, H3A 0B8, Canada;

[5]Quantitative Life Sciences, McGill University, Montreal, Canada

[6]Department of Biology, McGill University, Montreal, Canada

[7]Department of Materials Science and Engineering, University of Pennsylvania, Philadelphia, PA, USA, 19104;

*Corresponding Author: vshenoy@seas.upenn.edu

Supplementary Figures and Tables

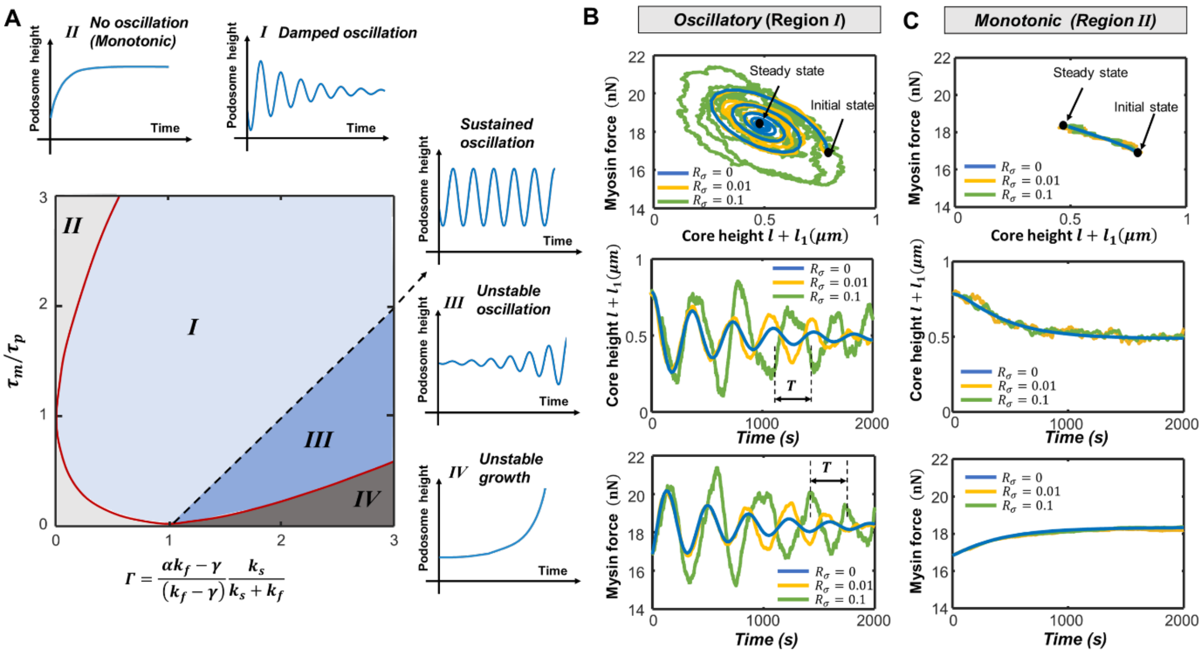

Supplementary Figure 1. Noise effects on podosome dynamics. (A) Phase diagram showing the different phases of protrusion dynamics based on timescale ratio τ_m/τ_p and the feedback parameter Γ. (B-C) The simulated (top panels) phase space of myosin force and core height plotted for (B) oscillatory region and (C) monotonic region. The simulated (middle panels) podosome core height and (bottom panels) myosin force plotted with time for (B) oscillatory region and (C) monotonic region. Lines with different colors indicate the Gaussian noises with different variance ratio R_σ. The variance ratio was defined as $R_\sigma = \sigma(\chi_p)/V_{p0} = \sigma(\chi_m)/F_{m0}$, where $\sigma(\chi_p)$ and $\sigma(\chi_m)$ denote the variances for actin polymerization and myosin dynamics, respectively. The oscillation period $T \approx 2\pi\sqrt{\tau_m\tau_p}$ is marked in (B). The myosin turnover time was set as $\tau_m = 50\, s$ to obtain the heavily damped oscillations for (B), while it was set as $\tau_m = 500\, s$ to obtain the monotonic growth for (C). All the other parameters are found in Supplementary Tables 1-2.

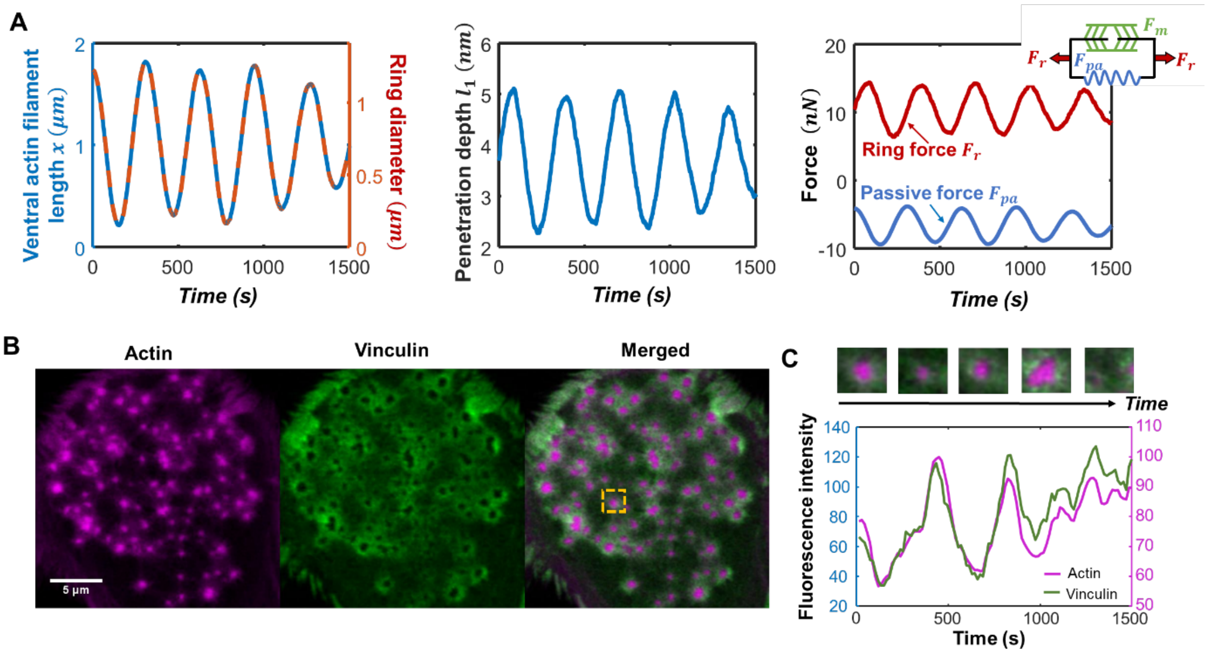

Supplementary Figure 2. Oscillatory behaviours in podosome core and ring. (A) The simulated (left panel) ventral actin filament length (blue line) and ring diameter (red line) plotted with time. (Middle panel) Simulated substrate displacement plotted with time. (Right panel) Simulated ring force (red line) and passive force of ventral actin filaments (blue line) plotted with time. (B) Images of a DC stained for actin (magenta) and vinculin (green). (C) The experimentally measured fluorescence intensity of actin (magenta) and vinculin (green) for a representative podosome plotted versus time. The insets show the time series for the representative podosome marked in panel (B).

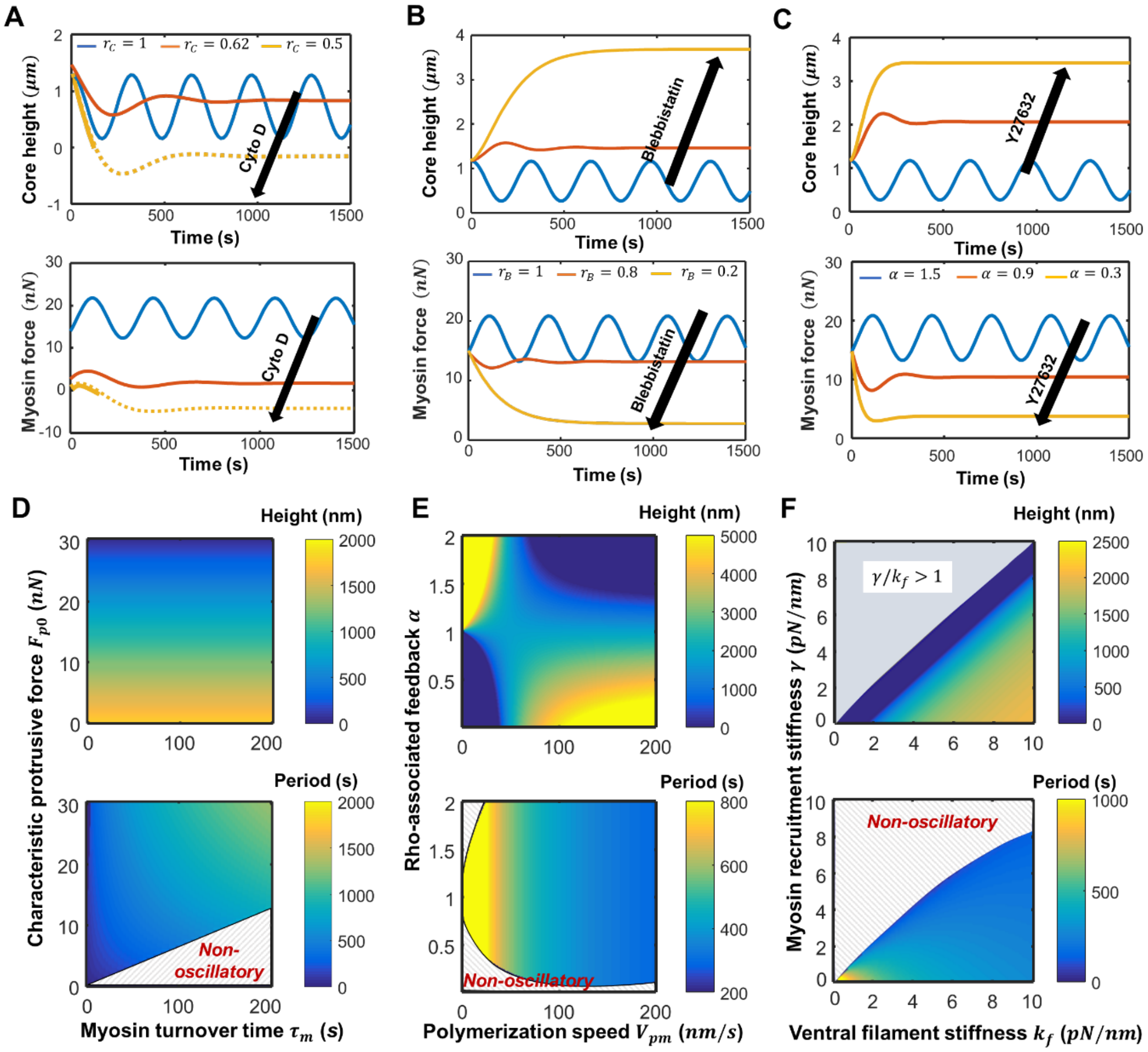

Supplementary Figure 3. Parameter sensitivity analysis. (A-C) The simulated (top panels) core height and (bottom panels) myosin force plotted with time for (A) cytochalasin D, (B) blebbistatin, and (C) Y27632 treatments. Blue lines indicate the control cases, while the red and yellow lines indicate the cases after the treatments. (D-F) The simulated (top panels) core height and (bottom panels) oscillation period plotted for (D) myosin turnover timescale and maximum protrusion force, (E) polymerization speed and Rho-associated feedback, and (F) ventral filament stiffness and effective stiffness for myosin contraction. The non-oscillatory regimes are marked with gray strips.

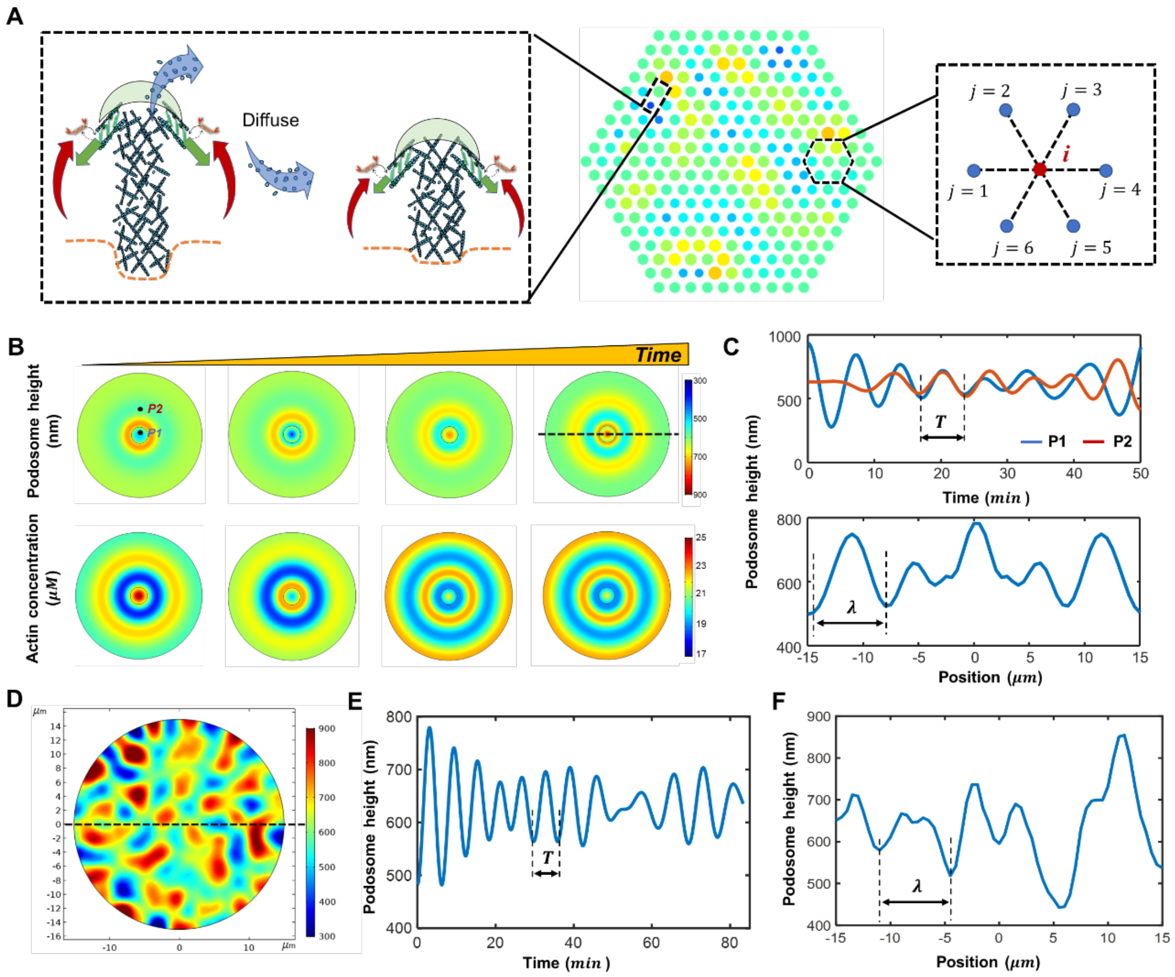

Supplementary Figure 4. The simulated spatiotemporal dynamics for radial and random waves. (A) Discrete model showing random wave patterns of podosome height. Individual podosomes are at a fixed separation $d_0 = 1.5\ \mu m$, and the dynamics of different podosomes are correlated through actin diffusion. (Left inset) Schematic for two neighbouring podosomes in the discrete model. (Right inset) The podosome i and its neighbouring podosomes j=1, 2, …,6. (B) (Top panels) The simulated podosome core heights and (Bottom panels) actin concentration in the cluster plotted for different instances of time. (C) (Top panel) The podosome height plotted with time for two representative points marked in panel (B). (Bottom panel) The podosome height plotted for different positions in the dashed cutting line in (B). (D) Simulated heights of a podosome cluster showing random waves. (E) The podosome height plotted with time for a representative point. (F) The podosome height plotted for different positions along the dashed cut line in (D). The wave periods T and wavelengths λ are labelled in (C), (E), and (F).

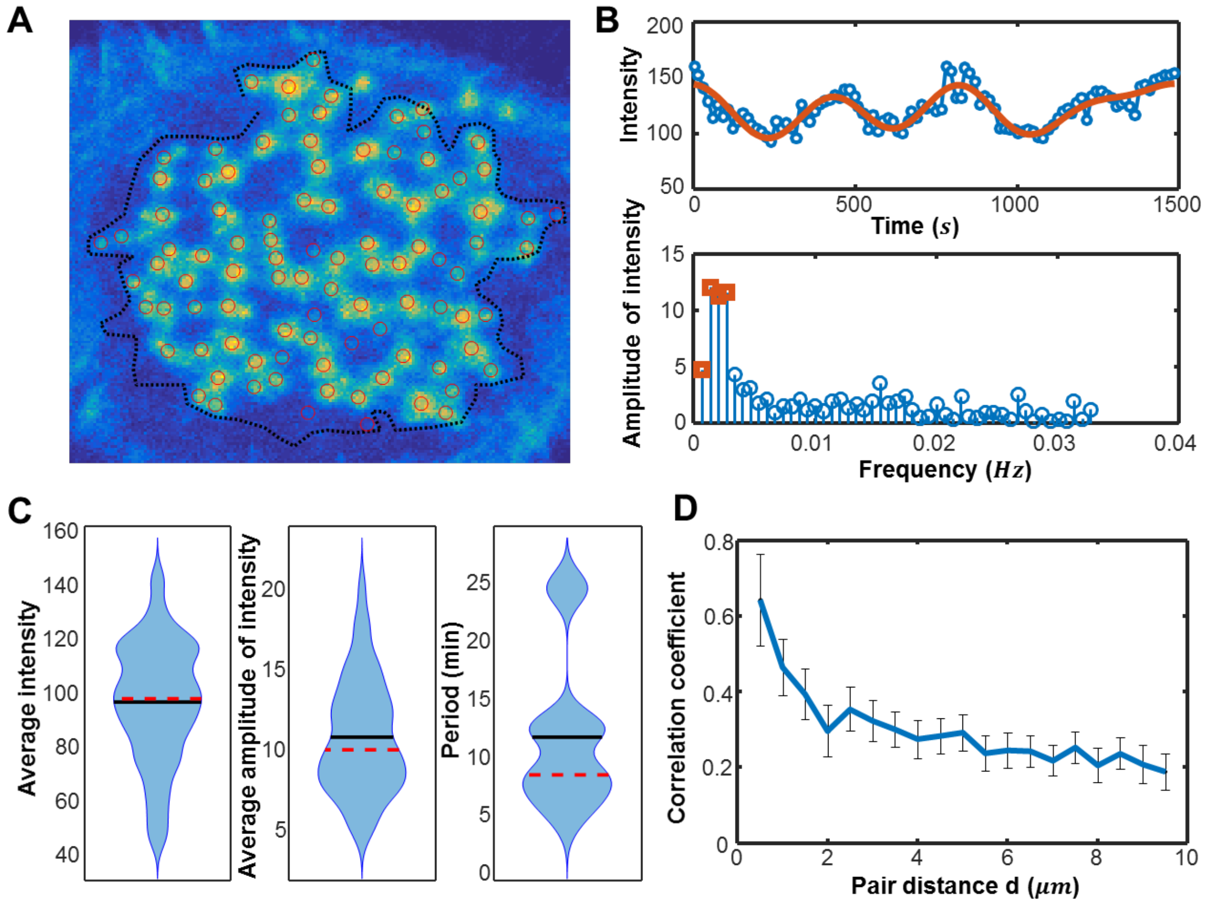

Supplementary Figure 5. Extraction of podosome oscillation periods and the correlation coefficient in a podosome cluster. (A) A representative LifeAct-RFP transfected DC. The red circles locate the podosome positions extracted using ImageJ, and the dashed line indicates the region of interest. (B) (Top panel) The fluorescence intensity of a representative podosome in (A) plotted with time. (Bottom panel) The frequency spectrum showing the amplitudes of intensity extracted by Fast Fourier Transformation (FFT) as a function of frequency. Blue dots and line represent the experimentally measured dynamics, and the red line in the top panel indicates the time-domain dynamics transformed from the largest four amplitude peaks in the spectrum (i.e., red markers in the bottom panel) using FFT. (C) Violin plots for (left panel) average fluorescence intensity, (middle panel) averaged amplitude, and (right panel) period for the representative DC shown in (A). Podosome number n=89. (D) The correlation coefficient plotted as a function of pair distance for the representative DC shown in (A). Podosome pair number n=3916. Data are presented as mean values +/- SEM.

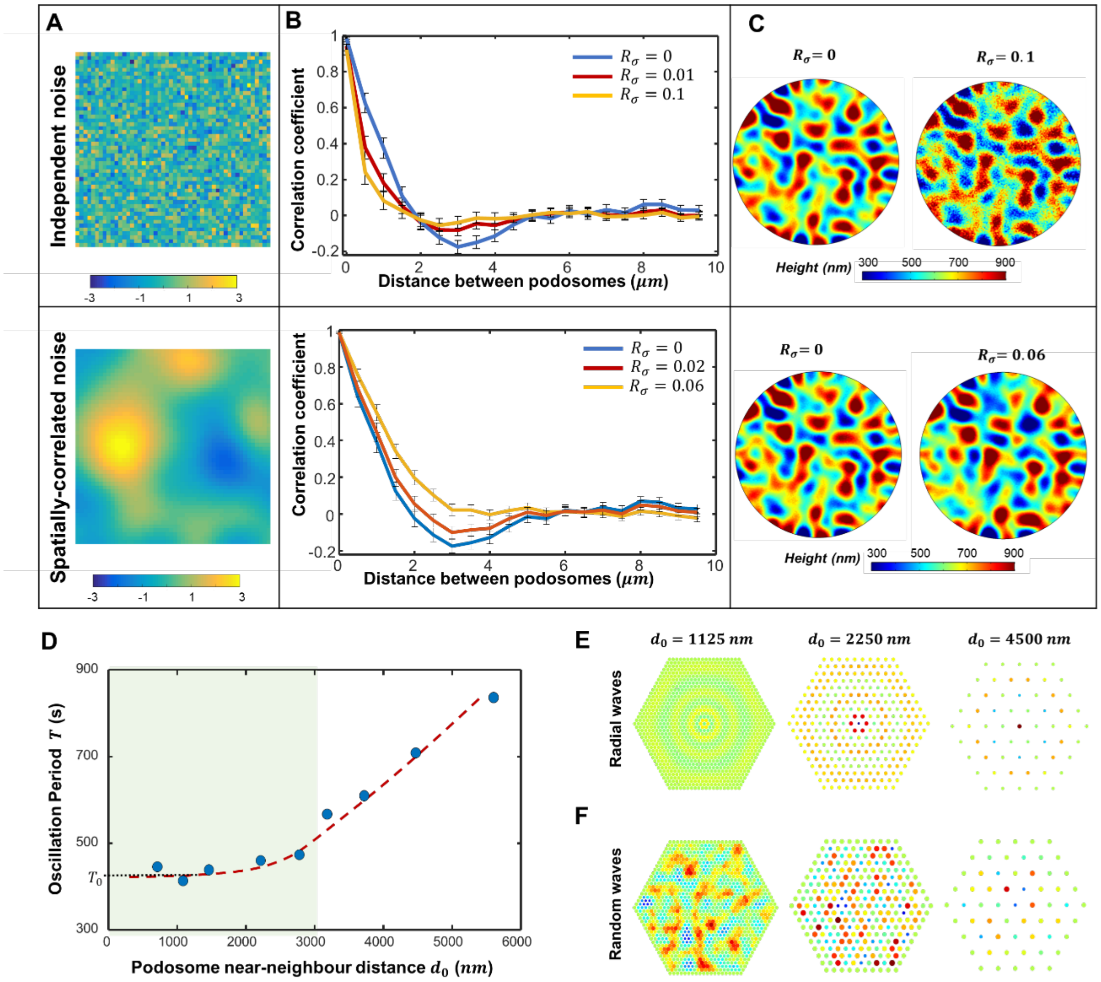

Supplementary Figure 6. The effects of noise and near-neighbour distance on simulated wave dynamics. (A) (Top panel) independent Gaussian noise and (bottom panel) spatially-correlated noise in a 2D plane. (B) The correlation coefficient plotted with the distance between podosomes for (top panel) independent Gaussian noise case and (bottom panel) spatially-correlated noise case. Lines with different colors correspond to different levels of noise, and the variance ratio was defined as $R_\sigma = \sigma/F_{m0}$, where σ denote the variances for the Gaussian noise of myosin force. n=18489 podosome pairs for each case. Data are presented as mean values +/- SEM. (C) The podosome height (left panel) without noise and (right panel) with noise for (top panel) independent Gaussian noise case and (bottom panel) spatially-correlated noise case. (D) The oscillation period T plotted as a function of podosome near-neighbour distance d_0 in our discrete model. Blue dots represent the mean oscillation periods extracted from the dynamics of podosomes simulated in the discrete model, while the red dashed line is the fitting line. Note that the oscillation period obtained from the continuum model is marked as T_0. (E-F) The discrete model of the podosome cluster with the near-neighbour distance (left panel) $d_0 = 1125\ nm$, (middle panel) $d_0 = 2250\ nm$, and (right panel) $d_0 = 4500\ nm$ for (E) radial waves and (F) random waves.

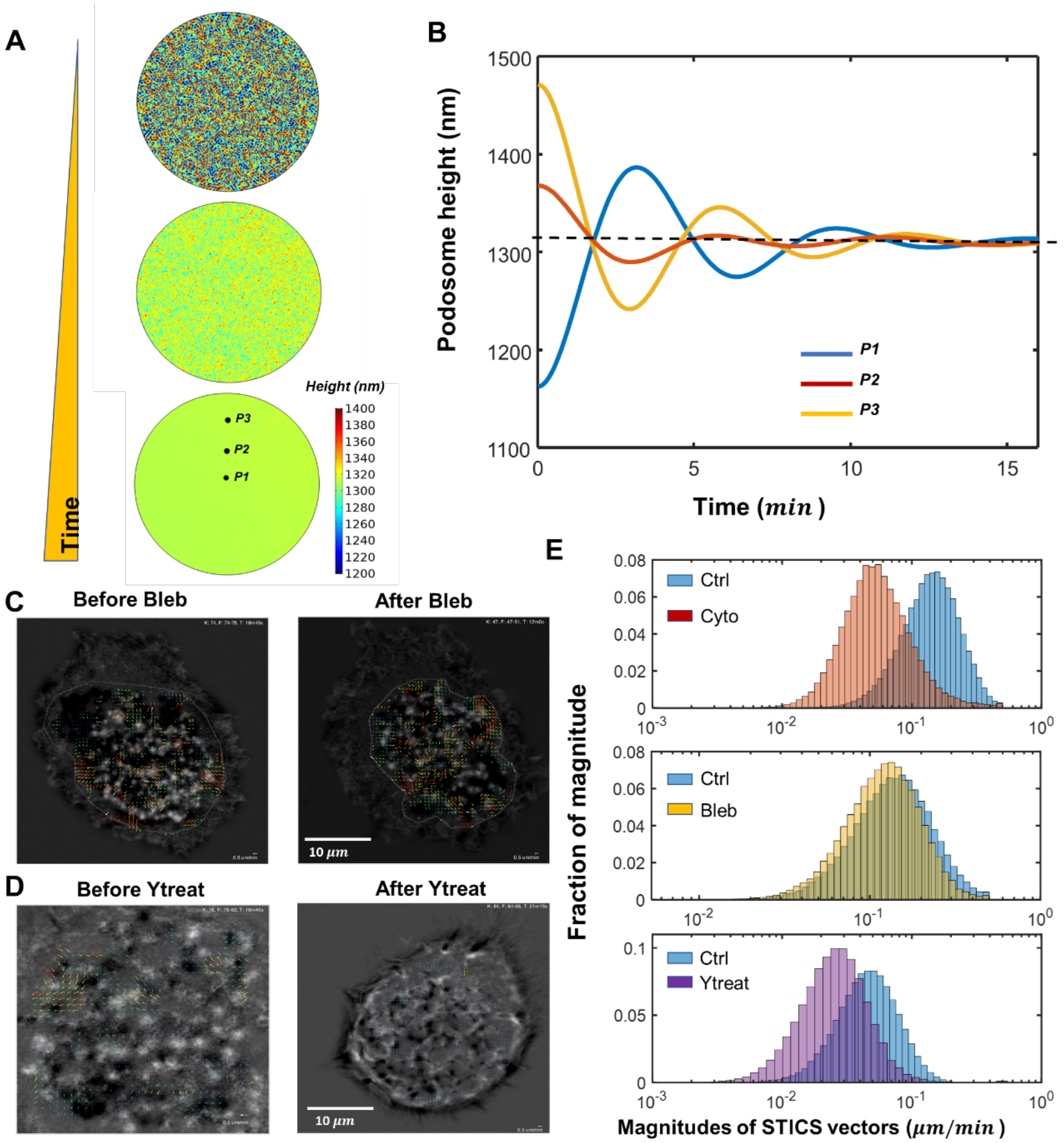

Supplementary Figure 7. Disrupted diffusion waves after the pharmacological treatments. (A) The simulated podosome core heights in the cluster plotted for different instances of time. (B) The ventral actin filament length plotted versus time for three representative points marked in (A). (C-D) Representative DC (left panel) before and (right panel) after adding (C) Blebbistain and (D) Y27632. DCs were transfected with LifeAct-GFP using confocal microscopy at 15 s intervals. Time series subject to STICS analysis were plotted as vector maps; the insets show the corresponding DCs without STICS analysis. The arrows indicate flow directions, and both the size and color denote the flow magnitude. (E) (Top panel) The fraction of velocity magnitudes measured by STICS for a same cell before (Ctrl, blue) and after cytochalasin D (Cyto, red) treatment; (Middle panel) The fraction of velocity magnitudes for a same cell before (Ctrl, blue) and after blebbistatin treatment (Bleb, yellow); (Right panel) The fraction of velocity magnitudes for control (Ctrl, blue) and Y27632 (Ytreat, purple) treatments.

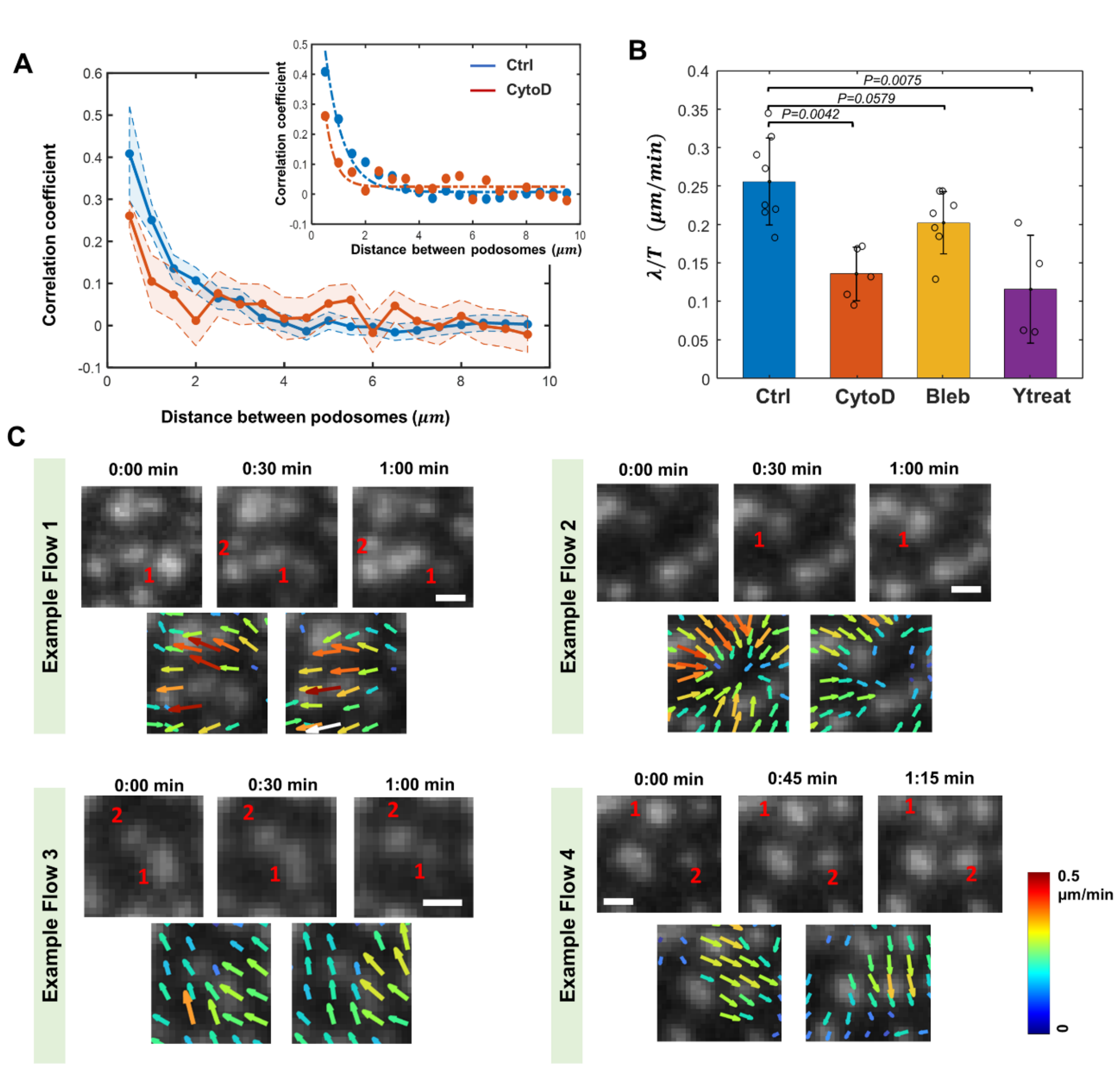

Supplementary Figure 8. The estimated wave speeds and STICS analysis. (A) The correlation coefficient plotted with the distance between podosomes for a representative dendritic cell before (Blue line and dots) and after cytochalasin D treatment (Red line and dots). Solid lines represent the mean and shade areas represent 95% confidence intervals. Inset: Data was fitted by the exponential decay function ($y = y_0 + e^{-x/\lambda_0}$; dashed lines) for a dendritic cell before and after cytochalasin D treatment. (B) The propagation speeds of waves λ/T for control (Ctrl, blue), cytochalasin D (CytoD, red), blebbistatin (Bleb, yellow), and Y27632 (Ytreat, purple) treatments. Data are presented as mean values +/-SD. ANOVA with Benjamini-Hochberg procedure was used. n = 8, 5, 7, 4 cells from left to right for 3 independent experiments. (C) Four representative examples of F-actin intensity flow regions with measured STICS vectors for Lifeact-RFP transfected dendritic cells. Top panels in each example: time series showing the dynamics of individual podosomes in the cluster, where the podosomes of interest are numbered. Bottom panels show STICS output vector maps for these regions in time. The arrows indicate flow

directions, and both the size and colour denote flow magnitudes. Example Flow 1: when the arrows are pointing towards left, podosome #2 becomes brighter while podosome #1 becomes less bright. Example Flow 2: when the STICS arrows are pointing towards the centre, podosome #1 in the centre position appears. Example Flow 3: when the STICS arrows are pointing left upward, podosome #2 becomes brighter while podosome #1 becomes less bright. Example Flow 4: when the STICS arrows are pointing right downward, podosome #2 becomes brighter while podosome #1 becomes less bright. Scale bar, 1 μm.

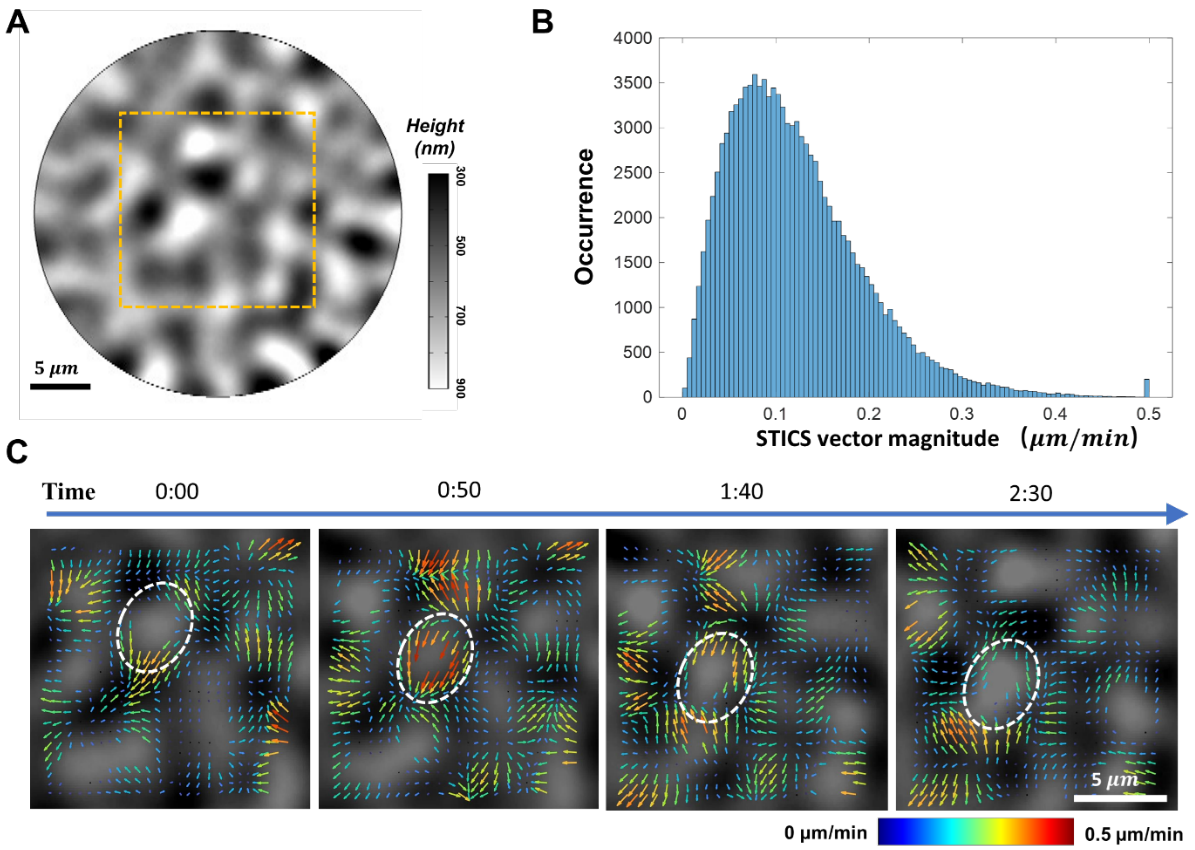

Supplementary Figure 9. STICS analysis on simulated podosome wave dynamics (A) The simulated heights for a representative podosome cluster. Scale bar, 5 μm. (B) The occurrence probability of flow velocity magnitudes is measured by STICS. (C) Time series of the indicated yellow rectangular area in panel A showing the wave-like dynamics in the podosome cluster. Time series subjected to STICS analysis are plotted as vector maps. The arrows indicate flow directions, and both the size and colour denote flow magnitudes. The circled regions with dashed line indicate the flow of the positions with large podosome heights (i.e., the propagation of the wave fronts). Scale bar, 5 μm.

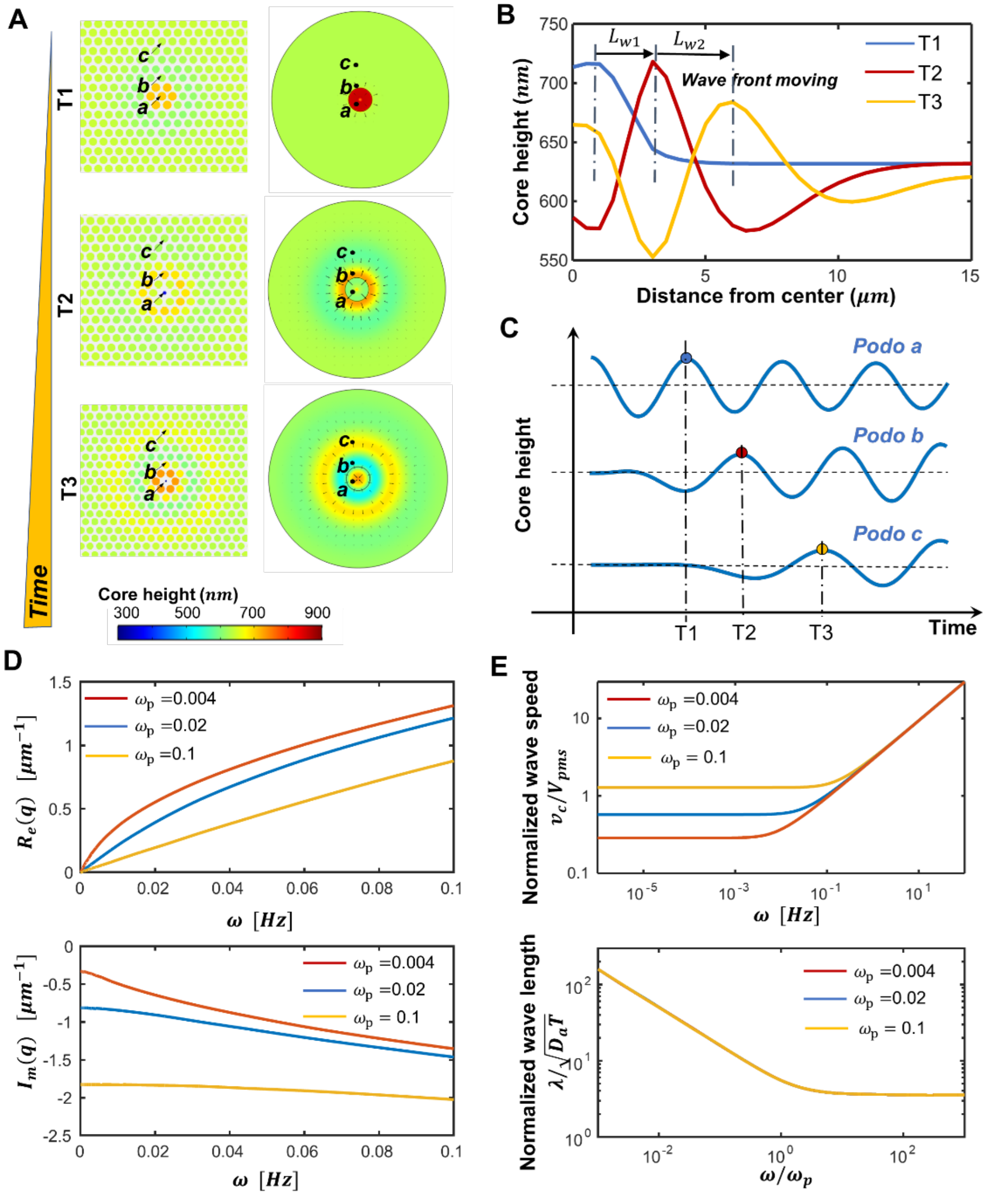

Supplementary Figure 10. Wave speeds and dispersion relation in simulations. (A) The simulated heights in the podosome cluster using (left panel) a discrete model of podosomes and (right panel) a continuum model showing radial wave patterns. (B) The core height plotted with the distance from the center for three different instances of time. (C) The core height plotted versus time for three representative podosomes marked in (A). Note that the curves in panels B and C are extracted from the continuum model, as the discrete and continuum models give similar results. (D) (Top panel) The real part $R_e(q)$ and (bottom panel) imaginary part $I_m(q)$ of the wavenumber plotted with different frequency ω with respect to different ω_p. (E) (Top panel) Normalized wave speed v_c/V_{pms} plotted with frequency ω. (Bottom panel) Normalized wave length $\lambda/\sqrt{D_aT}$ plotted with normalized frequency ω/ω_p with respect to different ω_p. Note that the curves for

normalized wave length (normalized by diffusion length) versus frequency overlap for different ω_p.

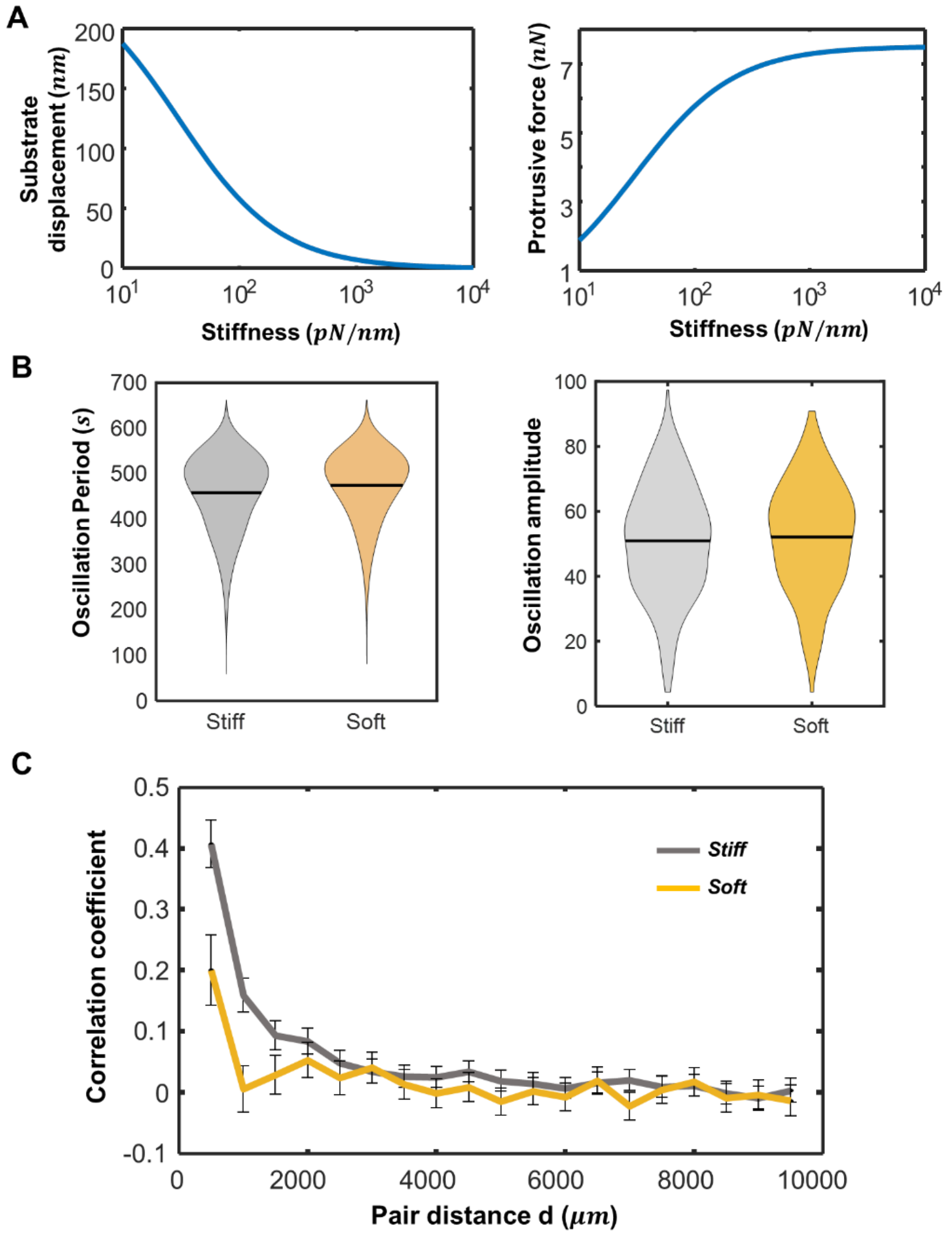

Supplementary Figure 11. Mechanosensing in a podosome cluster. (A) The simulated (left panel) substrate displacement and (right panel) protrusive force plotted for different levels of stiffness. (B) The extracted (left panel) oscillation periods and (right panel) amplitudes plotted for stiff and soft substrates. No significant difference was observed. (C) The correlation coefficient plotted as a function of pair distance for stiff and soft substrates. n=16704 and n=16844 podosome pairs for soft and stiff case. Data are presented as mean values +/- SEM.

Supplementary Table 1. Model variables

Symbols	Meaning	Value
F_p	Protrusive force of podosome core	~10nN
F_m	Myosin active contractile force	~20nN
c_a	G-actin concentration	~20μM
F_r	Ring force$F_r = F_p/\cos(\theta)$	~15nN
V_p	Polymerization speed$V_p = V_{pm}(1 - F_p/F_{sp0})$	~20-80nm/s
l	Core height above the undeformed substrate $l = x_r \cos(\theta)$	~0.2-1μm
l_1	Substrate displacement$l_1 = F_p/k_s$	~0.1-100nm
x_r	Ventral actin filament length $x_r = (F_r - F_m)/k_f + x_{r0}$	~0.4-2μm

Supplementary Table 2. Model parameters

Parameters	Meaning	Value	Reference
F_{p0}	Characteristic protrusive force	$20nN$	[1,2]
V_{p0}	Initial polymerization speed	$80nm/s$	[3,4]
V_d	Depolymerization speed	$50nm/s$	[5,6]
β	Sensitivity of polymerization speed to G-actin concentration	0.1-1 $nm \cdot s^{-1} \cdot \mu M^{-1}$	This article, refer to Supplementary Note 3.
k_s	Substrate stiffness	10-2000pN/nm	[3,7]
D_a	Diffusion coefficient	0.01-0.03 $\mu m^2/s$	This article, refer to Supplementary Note 3.
α	Rho-ROCK feedback parameter	1.5	[4,8]
γ	Effective stiffness for myosin contraction	$0.2pN/nm$	[4,8]
k_c, k_f	Stiffness for core F-actins and ventral F-actins	$10pN/nm$<br>$3.5pN/nm$	This article, refer to Supplementary Note 3.
c_{as}	Steady state G-actin concentration	$20\mu M$	[9,10]
χ_p, χ_m	Gaussian noise for actin polymerization and myosin recruitment	$\sigma(\chi_p) = 0.05V_{p0}$<br>$\sigma(\chi_m) = 0.05F_{p0}$	This article, refer to Supplementary Note 3.
θ	Angle between the ring ventral F-actins and the core	$\pi/4$	This article, refer to Supplementary Note 3.
τ_m	Characteristic time for myosin turnover	35 s	[11,12]
μ	Local actin concentration change per nanometer growth in podosome height	$0.01 - 0.1\ \mu M/nm$	This article, refer to Supplementary Note 3.
F_{m0}	Initial myosin force	1000 pN	[4]
x_{r0}	Initial length of ventral actin filaments	~2.8μm	This article, refer to Supplementary Note 3.
d_0	Podosome near-neighbour distance in the discrete model	1.5μm	[13,14]
R_0	Podosome cluster radius in the continuum model	15 μm	This article, refer to Supplementary Note 3.
ω_p	The effective actin production /consumption rate $\omega_p = \mu\beta V_d/V_{pms}$	$0.001 - 0.1$ rad/s	This article, refer to Supplementary Note 3.

Supplementary Note 1. Model development for wave-like dynamics in the podosome cluster

Core protrusion dynamics. Podosomes have a conical structure characterized by a protrusive actin-rich core, an adhesive ring, and ventral actin filaments that connect the core with the ring (Fig. 1A and 1B). In the podosome core, actin monomers (G-actin) continuously polymerize into actin filaments (F-actin) at the base while depolymerizing at the top of the core, so we can write the core height dynamics as the difference between the polymerization speed V_p and depolymerization speed V_d: $d(l + l_1)/dt = V_p - V_d$. Here $l + l_1$ is the total podosome height, where l is the core height above the undeformed substrate and l_1 is the displacement of the substrate (Fig. 1A). As the core F-actins assemble at the core base, generating a protrusive force F_p to resist polymerization[9,15], we can write the polymerization speed as $V_p = V_{pm}e^{-F_p/F_{sp0}} \approx V_{pm}(1 - F_p/F_{sp0})$, where V_{pm} is the maximum polymerization speed (in the absence of protrusive forces) and F_{sp0} is the characteristic protrusive force generated by the core. By assuming that F-actin depolymerizes at a constant speed V_d, the growth rate of the core height can be rewritten as:

$$\frac{d(l + l_1)}{dt} = V_{pm}\left(1 - \frac{F_p}{F_{sp0}}\right) - V_d + \chi_p(t)\,. \qquad (S1)$$

Here the Gaussian noise $\chi_p(t)$ accounts for fluctuations in the polymerization process. As the protrusive core deforms the underlying substrate and membrane, the substrate and membrane displacement can be expressed as $l_1 = F_p/k_s$ with k_s representing the stiffness of the substrate. When the polymerization speed balances with the depolymerization speed (i.e., $V_p = V_d$) and the noise $\chi_p(t)$ is neglected, we obtain the steady-state protrusive force $F_{ps} = F_{sp0}(1 - V_d/V_{pms})$. To account for larger protrusive forces on stiffer substrates revealed by the previous experiments[2], we write the characteristic stall force as $F_{sp0} = F_{p0}k_s/(k_c + k_s)$, where k_c is the core stiffness and F_{p0} is the characteristic protrusive force on rigid substrates. Microscopically, this larger protrusive force is due to a larger core size and more core F-actin on stiffer substrates. Thus, a higher substrate stiffness increases the steady-state protrusive force $F_{ps} = F_{p0}k_s/(k_s + k_c)(1 - V_d/V_{pms})$ while reducing the substrate displacements F_{ps}/k_s (Supplementary Figure 1A), in line with previous experimental measurements[2,13].

Force balance between ring and core. As the F-actin network assembles at the podosome core and generates a protrusive force F_p, myosin motors exert contractile forces on the ventral actin filaments to balance the core protrusive force and constrain core growth. To model the contraction

of actomyosin filaments, we adopt a two-element active contraction model[8], which consists of an active element (with contractile force F_m) in parallel with a passive elastic element (with stiffness k_f). The active element characterizes myosin contractility, while the passive (elastic) element represents the stiffness of the ventral actin filaments (Fig. 1B, inset i). Thus, the tensile force sustained by the ring F_r can be written as the sum of the active and passive forces:

$$F_r = F_m + k_f(x_r - x_{r0}), \qquad (S2)$$

where x_r and x_{r0} denote the current and initial length of the ventral actin filaments, respectively. Assuming that the cone-like structure of the podosome has a constant angle θ between the ventral actin filaments and the core, we have the geometric constraint and force balance as:

$$x_r cos(\theta) = l, \quad F_p = F_r \cos(\theta); \qquad (S3)$$

Note that the experimentally obtained high correlation between the F-actin and vinculin-related fluorescence signals (Supplementary Figure 2C) provides evidence that the angle θ is constant. Combining Eqs. (S1-S3), we can obtain:

$$\frac{F_{sp0}}{V_{pm}}\left(\frac{1}{k_f} + \frac{1}{k_s}\right)\frac{dF_p}{dt} + F_p = F_{sp0}\left(1 - \frac{V_d - \chi_p(t)}{V_{pm}}\right) + \frac{F_{sp0}}{V_{pm}}\frac{cos(\theta)}{k_f}\frac{dF_m}{dt}; \qquad (S4)$$

Mechanosensitive recruitment of myosin. The dynamics of active myosin contractility is determined by the processes of myosin recruitment (binding to VAFs) and turnover (unbinding from VAFs). Assuming there is a large cytoplasmic pool of myosin, of which the total myosin number is much larger than the bound myosin number. (i.e., $m_0 \gg m_b$), the dynamics of bound myosin is:

$$\frac{dm_b}{dt} \approx k_{on}^* m_0 - k_{off} m_b + \xi_m(t). \qquad (S5)$$

Here k_{on}^* and k_{off} are the rates for myosin recruitment and turnover, respectively, and $\xi_m(t)$ is a Gaussian noise term to represent the fluctuations in myosin number during myosin dynamics. As discussed in the first *Results* section, mechanosensitive biochemical pathways (Rho-ROCK signaling) reinforce myosin recruitment, while a large VAF displacement $x_r - x_{r0}$ reduces myosin recruitment (Fig. 1B). Therefore, we can write the effective binding rate of myosin as follows:

$$k_{on}^* = k_{on}^0 + \frac{\alpha_0 k_{on}^0}{f_b} F_r - \gamma_0 k_{on}^0 \frac{(x_r - x_{r0})}{x_{r0}}. \qquad (S6)$$

Here k_{on}^0 is the initial myosin binding rate (i.e., without signaling), while α_0 and γ_0 are

nondimensional parameters characterizing the impact on myosin recruitment from the Rho-ROCK pathway and VAF displacement, respectively. The active myosin force can be further calculated as $F_m = m_b f_b$, where f_b is the characteristic force per myosin. By substituting Eq. S6 and the relation $F_m = m_b f_b$ into Eq. S5, we can obtain the dynamics of myosin force as:

$$\tau_m \frac{dF_m}{dt} + F_m = F_{m0} - \gamma(x_r - x_{r0}) + \alpha F_r + \chi_m(t). \qquad (S7)$$

Here $\tau_m = 1/k_{off}$ is the characteristic time for myosin turnover, and $\alpha = \alpha_0 k_{on}^0 m_0 / k_{off}$ and $\gamma = \gamma_0 k_{on}^0 f_b m_0 / x_0 / k_{off}$ are two coefficients characterizing the effects of Rho-ROCK and VAF displacement on myosin recruitment, respectively. $\chi_m(t) = f_b \xi_m(t)/k_{off}$ represents the fluctuation in myosin dynamics, and $F_{m0} = m_0 f_b k_{on}^0 / k_{off}$ is the myosin force equilibrium constant when the signaling feedback is absent. Substituting Eqs. S2-S3 into Eq. S7, we obtain:

$$\tau_m \frac{dF_m}{dt} + \left(1 - \frac{\gamma}{k_f}\right) F_m = F_{m0} + \left(\alpha - \frac{\gamma}{k_f}\right) \frac{F_p}{cos(\theta)} + \chi_m(t). \qquad (S8)$$

Diffusion of G-actin. The above differential equations (Eqs. S4 and S7) yield the dynamics for individual podosomes in a cluster. To model the spatiotemporal collective dynamics, we consider the diffusion and exchange of actin between the podosomes within the cluster. Considering the G-actin concentration $c_a(x, y, t)$ in the *x-y* plane of the cluster at time t, the rate of concentration change is given by the diffusive flux and reaction (sources/sinks) of G-actin in the system within a control volume: $\partial c_a / \partial t = -\nabla \cdot \vec{J} + R_a$, where $\vec{J}$ is the total flux and R_a is a net volumetric source/sink for actin concentration, c_a. We can approximate the flux as $\vec{J} = -D_a \nabla c_a$ with the diffusion constant D_a based on Fick's first law. Assuming the podosome core has N_a actin filaments, the density of G-actin consumed per second through polymerization is $N_a V_p / \delta_a$, and the density of G-actin released per second through depolymerization is $N_a V_d / \delta_a$, with δ_a representing the half size of actin monomers. The reaction term R_a can be represented as the net rate difference between consumption and production, i.e., $R_a \approx -\eta N (V_p - V_d)/\delta_a$, where η represents the geometric coefficient with unit μM. It is convenient to introduce the factor $\mu = \eta N / \delta_a$ with the dimension $\mu M / \mu m$ that converts the length units of the podosome core into the concentration units of G-actin. Hence, we can write the diffusion-reaction equation for G-actin as:

$$\frac{\partial c_a}{\partial t} = D_a \nabla^2 c_a - \mu \cdot \left[V_{pm} \left(1 - \frac{F_p}{F_{sp0}}\right) - V_d \right]. \qquad (S9)$$

Note that a larger G-actin concentration increases the polymerization speed, that is $V_{pm} = V_{p0} + \beta c_a$, where a linear dependence of polymerization speed on actin concentration with sensitivity β is assumed for simplicity. We assumed no boundary flux, or $\vec{n} \cdot \nabla c_a = 0$, where $\vec{n}$ is the normal to the boundary surface, for G-actin in our simulations. Overall, Eqs. S4, S8, and S9 correspond to the governing equations (Eqs. 5-7) for podosome dynamics in a cluster. All the model parameters are summarized in Supplementary Table 2, and the discussion on the parameter selection can be found in Supplementary Note 3.

Supplementary Note 2. Discrete and continuum approaches for solving the equations

To solve the ordinary differential Eqs. 4-5 coupled with the diffusion Eq. 6, we applied both the discrete and continuum approaches. In our discrete model, we first generate a triangular lattice for the podosome cluster, such that each node represents an individual podosome. The dynamics of each podosome in the cluster is governed by the polymerization-associated protrusion (Eq. 4) and signaling-associated myosin recruitment dynamics (Eq. 5). Meanwhile, G-actin can freely diffuse within the podosome cluster (Eq. 6) and affects the maximum polymerization speed (in Eq. 4) through the relation $V_{pm} = V_{p0} + c_a\beta$. As the distance between neighbouring podosomes is assumed as distance d_0, we can calculate the divergence of the G-actin concentration gradient at the ith podosome as:

$$\nabla^2 c_a^{i,0} = \sum_{j=1}^{3} \frac{c_a^{i,j} + c_a^{i,j+3} - 2c_a^{i,0}}{d_0^2}, \qquad j = 1,2,3. \qquad (S10)$$

Here the superscript $c_a^{i,j}$ indicates the j th neighbouring podosome for the podosome i (Supplementary Figure 4A), and we use $c_a^{i,0}$ to denote the actin concentration at the i th podosome. A podosome that is not in the boundary has six neighbouring podosomes ($j = 1, 2, 3, ..,6$). For those podosomes that are in the boundary, we set the normal component of the gradient $\vec{n} \cdot \nabla c_a^i = 0$ for the assumption of no flow across the boundary. We can also take the continuum limit of the discrete model, where we treat the podosome height in the cluster as a continuous variable. This continuum model (Eq. S9) can be considered as a generalization for the discrete cases with podosome neighbouring distance below diffusion the length scale ($d_0 < \sqrt{D_a T}$), and the reasoning can be found in Supplementary Note 3—Effects of near-neighbour

distance on podosome dynamics. The continuum model can be solved along with the differential Eqs. 5-7 using the Mathematics Module in COMSOL. In the simulation, the initial value of the G-actin concentration is chosen to be uniform at its steady-state limit, c_{as}, while the initial values for protrusive/myosin forces are varied ~20% from their steady-state values (F_{ps}, F_{ms}). Note that the initial conditions do not affect the oscillatory or monotonic growth of individual podosomes and collective wave-like dynamics.

Supplementary Note 3. Parameter justification for the model

Timescales in podosome clusters: Following previous theoretical studies[16,17], we adopted the timescale $\tau_m \sim 35s$ for myosin turnover. This timescale τ_m for myosin recruitment or turnover here does not necessarily describe the single myosin turnover dynamics (which happens much faster at ~1 s), as we do not distinguish whether changes in the myosin kinetics are the result of alterations in myosin turnover, changes in available cortical binding sites, or intracellular flow of myosin. The protrusion timescale for the podosome core is calculated as $\tau_p = \frac{F_{sp0}}{V_{pms}}(\frac{1}{k_f} + \frac{1}{k_s})(1 - \frac{\gamma}{k_f}) \sim 50\ s$ based on the parameter values in Supplementary Table 2, and both a larger substrate stiffness k_s and a larger polymerization speed V_{pms} can reduce the protrusion timescale. When the protrusion timescale and myosin turnover timescale are comparable ($\tau_m/\tau_p \sim 1$), podosomes begin to oscillate, and the estimated oscillation period $2\pi\sqrt{\tau_m \tau_p}$ is approximately 400 s, in line with our experimental measurement (Fig. 4F).

Length scales in podosome clusters: In our model, we simplified a single podosome as a conical shape with a constant half apex angle of $\theta = \pi/4$. We chose this setting for two reasons. First, previous experiments[18] have shown that the podosome core height is 400-900 nm, while the ring radius is measured to be approximately 500~900 nm [2,13]. Hence, the half apex angle is set as $\theta = \pi/4$ such that the podosome core height h and ring radius r are equal, i.e., $h = r \cdot \tan\left(\frac{\pi}{4}\right)$. Second, the high correlation between the actin and vinculin fluorescence intensity (Supplementary Figure 2C) can be taken as evidence for the constant apex angle. Microscopically, similar to the Arp 2/3 complex that yields a constant branch angle, this fixed angle may come from the structure of the protein that connects the core actin and ventral F-actin, although there are no reports on the connecting proteins between the core F-actin and ventral F-actin. Previous experiments have

quantified the distance between individual podosomes as ~1.5 μm[19]. Considering $N_p = 100$ podosomes in a cluster, we can estimate the podosome cluster size as approximately $R_0 \sim d_0\sqrt{N_p} \approx 15\ \mu m$; this value can also be verified from the experimental images (Fig. 2A and 3C), which shows the cell diameter as $\sim 30\ \mu m$.

Forces and stiffnesses in the podosome system: In our podosome growth model, the protrusive force F_p generated by the core actin at a steady state is governed by the ratio of depolymerization and polymerization speed V_d/V_{pms} as well as the ratio between substrate stiffness and core stiffness k_c/k_s, i.e., $F_{ps} = F_{p0}k_s/(k_c + k_s)\left(1 - V_d/V_{pms}\right)$, and its magnitude is $F_{ps} \sim 10\ nN$. This force magnitude also agrees with the previous measurement using protrusive force microscopy as well as previous theoretical modelling[1,20]. Meanwhile, the protrusive force is balanced by the forces sustained by the ring and VAFs, which consists of the myosin contractile force with a magnitude of $F_m \sim 20\ nN$ and the passive compressive force of VAFs with a magnitude of $F_{pa} \sim 10\ nN$ (Supplementary Figure 2A). Note that, for simplicity, we assume the substrates to be linear elastic materials since the deformations are relatively small. The substrate stiffness is chosen in the range of 10-2000 pN/nm based on our previous studies[21]. Previous experiments[18] have quantified the Young's modulus of podosome cores to be ~50 kPa. The core stiffness can be estimated as $\sim 50\ kPa \times 0.2\ \mu m = 10\ pN/nm$ by assuming the podosome core has a circled cross-section with a radius of 0.2 μm.

Dynamics of actin: To estimate the parameters that govern the G-actin production/consumption rate (e.g., μ and ω_p), we consider that the podosome core has a circled cross-section with a radius of 0.2 μm and hence a cross-section area $A \approx \pi \times (0.2\ \mu m)^2 \approx 0.12\ \mu m^2$. As previous work[9] has estimated the line density of actin filaments in filopodia to be ~50 filaments per micrometer, we can estimate the planar density of actin filaments as $\rho \sim (50\ \mu m^{-1})^2 = 2500\ \mu m^{-2}$ and the number of actin filaments as $N_a = \rho A \approx 300$. Considering the half size of actin monomer $\delta_a \approx 2.7\ nm$ as estimated previously[6,9], the podosome core releases $1nm \times N_a/\delta_a \sim 10^2$ actin monomers per nanometer change in the core height. As the cell volume is $V_{cell} \sim 10^3 - 10^4\ \mu m^3$, the geometry coefficient η, corresponding to the molar concentration change when a single actin monomer is released in the cytosol, can be estimated as $\eta \approx 1/V_{cell}/(6.02 \times 10^{23}\ mol^{-1})\ \sim 10^{-4} - 10^{-3}\ \mu M$. Hence, the G-actin consumption rate per core height change is $\mu = \eta N_a/\delta_a \sim 0.01 - 0.1\ \mu M/nm$. Considering the G-actin assembly rate $k_{on}^{actin} \sim 10\ \mu M^{-1} \cdot$

s^{-1} as estimated previously[10], the sensitivity of polymerization speed to G-actin concentration $\beta = k_{on}^{actin}\delta_a/N_a$ is in the range of $\sim 0.1 - 1\, nm \cdot \mu M^{-1} \cdot s^{-1}$. Thus, the effective actin production/consumption rate is estimated as $\omega_p = \mu\beta V_d/V_{pms} \approx 0.001 - 0.1\, s^{-1}$. For our simulations for control cases, we adopt $\mu = 0.04\, \mu M/nm$, $\beta = 0.4\, nm \cdot \mu M^{-1} \cdot s^{-1}$, and $\omega_p \approx 0.01\, s^{-1}$.

To further estimate the diffusion constant D_a, we note that the podosome oscillation period is $T \approx 400\, s$ and the experimentally measured wavelength scale is $\lambda \approx 3\, \mu m$; hence, we can estimate the diffusion constant D_a in the magnitude of $0.02\, \mu m^2/s$ based on the relation $\lambda \sim \sqrt{D_a T}$ predicted by our model. This small diffusion constant can be attributed to the relatively slow in-membrane diffusion of integrin and adaptors[22], as core growth and F-actin assembly require the recruitment of ring components to anchor the base to substrates. In other words, the diffusion constant is an effective constant for podosome components, and the reaction-diffusion process (Eq. 7) can be considered as a combined or effective dynamics of the different podosome components, including F-actin, integrin, and vinculin.

Effects of near-neighour distance on podosome dynamics: To see the distance effect on podosome dynamics, we conducted simulations by varying the near-neighour distance d_0 in our discrete model. Our simulations show that both the oscillation periods of individual podosomes (Supplementary Figure 6C) and wave patterns (Supplementary Figure 6D-E) obtained from our discrete model are in accordance with the results from the continuum model (Fig. 4D and 4F) when the near-neighbour distance d_0 is below 3 μm. Beyond this critical distance, the podosome oscillation periods increase, and the wave patterns become obscure (Supplementary Figure 6D-E). This critical near-neighour distance is the diffusion length scale $\sqrt{D_a T_0} \approx 3\, \mu m$, where T_0 is the oscillation period of podosomes when their neighbouring distance is small (discrete model with $d_0 \leq 3\, \mu m$ or continuum model). As the near-neighour distance exceeds the diffusion length scale, the time taken for G-actin to diffuse between the neighbouring podosomes becomes larger than the oscillation period T_0 (i.e., $d_0^2/D_a > T_0$); the oscillation of one podosome could affect its neighbouring podosomes through G-actin diffusion only after a long-time delay d_0^2/D_a, which slows down the oscillations and leads to extended oscillation periods. When $d_0^2/D_a < T_0$, the time delay due to diffusion is relatively small, and the podosome oscillations are not affected. Hence, we can conclude that our continuum model, which yields a spatial average of the podosome

heights, can be considered to be a generalization for all the discrete cases with the near-neighour distance below the diffusion length scale ($d_0 \leq \sqrt{D_a T_0}$).

Mechanosensitive signaling feedback: The feedback parameter group $\Gamma = \frac{\alpha k_f - \gamma}{(k_f - \gamma)} \frac{k_s}{k_s + k_f}$ mainly contains three parameters: α, k_f, and γ. The parameters α and γ characterize the effects of Rho-ROCK and VAF displacement on active forces (myosin contractility), while k_f is the stiffness that characterizes the VAF displacement on the passive force. As the myosin contractility at steady state $F_{ms} = \frac{k_f}{k_f - \gamma}(F_0 + \left(\alpha - \frac{\gamma}{k_f}\right) \frac{F_{ps}}{cos(\theta)})$ is always positive, we have the condition $\gamma/k_f < 1$. In addition, as the feedback groups $\Gamma = \frac{\alpha k_f - \gamma}{(k_f - \gamma)} \frac{k_s}{k_s + k_f}$ are positive, we have the condition $\alpha > \gamma/k_f$. Here, in our model, we have $\gamma/k_f \approx 0.06$, indicating that the influence of ventral actin filament length change on the active force is negligible compared to its influence on the passive force. The values of these three parameters (α, k_f, and γ) are chosen based on our previous publications[4,8].

Noise effects on individual podosome dynamics: Two Gaussian noise terms $\chi_p(t)$ and $\chi_m(t)$ were applied to account for the random fluctuation in actin polymerization and the myosin recruitment dynamics. The Gaussian noises are generated by MATLAB *randn* function $\chi_m(t)$, $\chi_p(t) \sim N(0, \sigma)$, where their standard deviation is estimated at 1%-10% of the characteristic protrusive force ($\sigma(\chi_m) = 0.05F_{p0} \sim 5000\, pN$) and initial polymerization speed ($\sigma(\chi_p) = 0.05V_{p0} \sim 5\, nm/s$), respectively. These noises added to the system do not change the steady-state lengths significantly; however, they influence the dynamics differently based on the different phase regions. In oscillation regime I (Fig. 1C and Supplementary Figure 1A), the podosome protrusion system works as a "stochastic amplifier", amplifying the small noise signal into a large oscillation in protrusion length. This is because the random fluctuations constantly deviate the podosome system away from its steady state, initiating excursions in the phase space of core height and myosin force. Eventually, these noise signals can sustain podosome persistent oscillations with a random amplitude but a fixed oscillation period $\sim 2\pi\sqrt{\tau_m \tau_p}$ (Supplementary Figure 1B). In the monotonic growth regime II, although the noise can imply small fluctuations in the podosome heights, it cannot give oscillatory behaviours with fixed periods (Supplementary Figure 1B-C). In experiments, the environmental noises can cause time-dependent variations even for monotonically growing protrusions, making it difficult to differentiate the oscillatory and non-

oscillatory behaviours in experiments. This is also the reason that we introduced the amplitude ratio, defined as $r_a = A_{osc}/\bar{R}$, to quantify the intensity of oscillation in podosome dynamics.

Noise effects on collective dynamics of podosomes: In our model for the collective dynamics of podosomes, we first applied the independent Gaussian noises (Supplementary Figure 6A top panel) to the individual podosomes of cluster (i.e., the nodes in the continuum model). Note that, for simplicity, we only applied the Gaussian noises to the dynamics of myosin force in our simulations. Our simulations show that noise does not influence the wave patterns (Supplementary Figure 6C top panel); A higher level of independent noise decreases the degree of anti-correlation while also reduces the positive correlation in our correlation coefficient plots (Supplementary Figure 6B top panel). As noise signals in vitro should be strongly correlated in short distances and becomes uncorrelated over large distances, we next applied the spatially-correlated noise to the system. To generate this spatially-correlated noise (Supplementary Figure 6A bottom panel), we applied a k-dimensional random vector $\boldsymbol{X} = (X_1, \dots, X_k)$ with a multivariate Gaussian distribution $\boldsymbol{X} \sim \mathcal{N}_k(\boldsymbol{\mu}, \boldsymbol{\Sigma})$, where its mean vector $\mu_i = E[X_i]$ is a zero vector and the covariance matrix $\Sigma_{i,j} = \mathrm{Cov}[X_i, X_j]$ specifies the correlation between different vector components. Considering the random variable of vector X_i represents the noise signal for the podosome i in the cluster, the correlation coefficient between podosome i and podosome j decays with their distance d_{ij}. Hence, we can write the covariance matrix as $\mathrm{Cov}[X_i, X_j] = \sigma e^{-\left(\frac{d_{ij}}{d_c}\right)^2}$ with d_c representing a characteristic decay length, such that the correlation decays with the distance exponentially. When $i = j$, we get the distance $d_{ij} = 0$ and the variance $\mathrm{Cov}[X_i, X_i] = \sigma$. After incorporating this spatial-correlated noise into our continuum model, we found that it could remove the negative correlation while elevate the positive correlation in the correlation coefficient plot (Supplementary Figure 6B bottom panel). This is different from the previous spatially-uncorrelated noises where the positive correlation coefficients get decreased (Supplementary Figure 6B top panel). Clearly, the spatially-correlated noise is more reasonable in our podosome system, as the correlation coefficient plot (Supplementary Figure 6B bottom panel) is more resemble to the plot from experiments (Fig. 4G). In addition, previous studies have also used the spatially-correlated noise in the biological systems, including DNA charge transfer[23] and neuronal firing[24,25].

Sensitivity analysis: Heatmaps for the podosome core height at steady-state and oscillation periods (non-oscillatory regimes are marked with gray strips) were plotted as a function of

different key parameters (Supplementary Figure 3D-F). In our simulations, a larger characteristic protrusive force F_{p0} reduces the podosome steady-state height while increasing the oscillation periods, and the myosin turnover timescale τ_m does not affect the podosome heights at a steady state but increases the podosome oscillation periods (Supplementary Figure 3D). A smaller Rho-associated feedback parameter α reduces the myosin contractility at the VAFs, leading to larger podosome heights. Meanwhile, a larger polymerization speed V_{pm} can increase the podosome core heights when Rho-associated feedback is small (α<1). However, for a larger Rho-associated feedback parameter (α >1), larger polymerization can reduce the podosome heights (Supplementary Figure 3E). This negative regulatory effect is because a large polymerization speed can cause large protrusive forces and hence larger ring forces, triggering more myosin recruitment through the Rho-ROCK pathway, which eventually constrains podosome growth more. Furthermore, a larger VAF stiffness increases the podosome heights, as it becomes more difficult to compress VAFs and constrain height growth, while a larger myosin recruitment stiffness reduces the heights as more myosin is recruited to exert contraction (Supplementary Figure 3F). The regulation effects of key components are further examined using different inhibitor treatments (Cytochalasin D, Y27632, and Blebbistatin), which can also be found in the heatmaps. For Bleb or Y27632 treatments, the decrease in parameters, including α and γ, increases the podosome height and causes podosomes to undergo non-oscillatory dynamics (Supplementary Figure 3D). Cyto D treatment disrupted the polymerization in both the core actin and ring VAFs (i.e., reduced V_{pm}, α, and k_f), podosome heights were reduced after the treatments, and the oscillations are inhibited (Supplementary Figure 3D-F).

Supplementary Note 4. Model predicts individual podosome dynamics in response to different pharmacological treatments

First, we studied how the perturbation of signaling-associated myosin dynamics can affect podosome dynamics. To study the influence of the Rho-ROCK pathway on the podosome, we can simply reduce the signaling-associated parameter α. In the phase diagram (purple arrow in Fig. 1C), the reduced feedback parameter $\Gamma = \frac{\alpha k_f - \gamma}{(k_f - \gamma)} \frac{k_s}{k_s + k_f}$ leads to non-oscillatory behaviours. By reducing the parameter α, our simulations indeed show that the oscillatory growth of podosomes

is inhibited (Supplementary Figure 3C), in agreement with our experiments (Fig. 2B and 2C). Next, to study the influence of Blebbistain (Bleb) treatment on podosome dynamics, we consider that myosin dynamics slow down after the treatment, corresponding to a larger myosin turnover time τ_m. Meanwhile, Bleb can reduce signaling-associated myosin recruitment and hence the feedback parameter Γ, which can be reflected in the phase diagram (Fig. 1C) using the yellow arrow. Therefore, we reduced the myosin-associated parameters (i.e., F_{m0}, α, and γ) by multiplying the ratio r_m ($r_m < 1$) while increasing the myosin turnover timescale by multiplying the ratio $1/r_m$ in our model. Similar to Y27632 treatments, we found that the oscillations in the core and ring were inhibited after treatments (i.e., reduced r_m in Supplementary Figure 3B), in agreement with our experiments (Fig. 2B). In addition to the changes in oscillatory patterns, our simulations show that the time-averaged core heights increase after either the Y27632 or Bleb treatment (Supplementary Figures 3B-C), as the VAF and myosin apply less contractility to restrict the core growth. Interestingly, our previous work also shows the increased fluorescence intensity of F-actins after Bleb treatments, which again validates our model predictions.

We next assessed how the inhibition of actin polymerization with cytochalasin D (Cyto D) affects podosome dynamics. The disruption of F-actins not only influences actin network assembly in the core but also affects myosin contractility at the ring, since fewer myosin motors are recruited to VAFs after F-actin disruption. In our model, both the polymerization speed V_{pm}, VAFs' passive stiffness k_f, and signaling-associated parameters α are decreased when F-actins are disrupted by Cyto D. Hence, the core protrusion timescale $\tau_p = \frac{F_{sp0}}{V_{pms}}(\frac{1}{k_f} + \frac{1}{k_s})(1 - \frac{\gamma}{k_f})$ increases, signaling feedback groups Γ decrease, and podosomes undergo non-oscillatory growth based on our phase diagram (red arrow in Fig. 1C). To further quantify the inhibition effects of Cyto D, we defined a parameter ratio r_c ($r_c < 1$) that is multiplied by the polymerization speed V_p, VAFs' passive stiffness k_f, and the signaling-associated parameters α in our model. We found that impaired F-actin inhibits the oscillatory growth in the core height (Supplementary Figure 3A). In addition to the changes in oscillatory patterns, our simulations show that Cyto D treatment significantly reduces the core heights and ring forces, which subsequently inhibits force-mediated adaptors, i.e., vinculin and paxillin. Interestingly, our previous work has also shown that the fluorescence intensities of F-actin, vinculin, and paxillin are significantly reduced after Cyto D treatments, in agreement with our simulations.

Supplementary Note 5. Linear perturbation for the podosome cluster dynamics system

For the linear stability analysis stated in Methods section, we applied a small perturbation to the individual podosome growth system (Eqs. 5-6) and G-actin diffusion-reaction system (Eq. 7) separately. In addition, we can apply a small perturbation of δF_{ps}, δF_{ms}, $\delta c_{as} \sim e^{i\omega t+iqr}$ to the steady state (F_{ps}, F_{ms}, c_{as}) of the entire system of podosome cluster dynamics (Eqs. 5-7) and obtained the relation between angular wavenumber q and angular frequency ω for the fully coupled system:

$$-\omega_p(1+i\omega\tau_m)+(D_a q^2+i\omega+\omega_p)((1+i\omega\tau_m)(1+i\omega\tau_p)-i\omega\tau_p\Gamma)=0, \qquad (S11)$$

where τ_m, τ_p, and Γ are the myosin turnover timescale, protrusion timescale, and signaling feedback, respectively. We should note that the G-actin consumption rate ω_p controls the connection between individual podosome oscillation and spatial wave-like dynamics. When $\omega_p \to 0$, the solution for Eq. S11 is $(1+i\omega\tau_m)(1+i\omega\tau_p)-i\omega\tau_p\Gamma=0$ or $q^2=-i\omega/D_a$. The former expression $(1+i\omega\tau_m)(1+i\omega\tau_p)-i\omega\tau_p\Gamma=0$ yields the same eigenvalues shown in Eq. 8 in Methods that applied stability analysis to individual podosome systems, while the latter solution $q^2=-i\omega/D_a$ gives the classical diffusion wavenumber. As either one can be the solution of Eq. S11, the individual podosome dynamics and spatial diffusion waves in podosome heights are uncorrelated. Hence, although we observed wave-like patterns in the actin concentration in our simulations with $\omega_p \to 0$, oscillations of heights in individual podosomes do not form wave-like dynamics. For our simulations with parameter values stated in Supplementary Table 2, we can approximate the q-ω relation as:

$$q^2=-\frac{i\omega+\omega_p}{D_a}\left[1-\frac{\omega_p(1+i\omega\tau_m)}{[(1+i\omega\tau_m)(1+i\omega\tau_p)-i\omega\tau_p\Gamma](iw+w_p)}\right]\approx-\frac{i\omega+\omega_p}{D_a}. \qquad (S12)$$

Therefore, the wavelength can be written as $\lambda=\frac{2\pi}{|\mathcal{R}_e(q)|}=\left(\frac{D_a^2}{\omega_p{}^2+\omega^2}\right)^{\frac{1}{4}}\frac{2\pi}{cos(\varphi/2)}$, where we have the augment $\varphi=arctan(\omega/\omega_p)-\pi$. In addition, similar to classical diffusion waves, the chemo-mechanical diffusion waves are heavily damped, as the magnitudes of the real part $\mathcal{R}_e(q)$ and imaginary part $\mathcal{I}_m(q)$ of the wave vector are comparable (Supplementary Figure 10D). The skin

or penetration depth that characterizes the damping effect is $\lambda_s = \frac{1}{|\mathcal{I}_m(q)|} = \left(\frac{D_a^2}{{\omega_p}^2+\omega^2}\right)^{\frac{1}{4}} \frac{1}{sin(\varphi/2)}$. The phase speed of the waves can be written as $v_c = \omega/\mathcal{R}_e\,(q) = \lambda/T$. Our model predicts that a large actin consumption rate ω_p increases the wavelength and wave speeds (Supplementary Figure 10E), which validates our experimental results that wave speeds increase for podosomes on stiffer substrates (Fig. 5F).

When analysing the wavelength and speed using the above linear perturbation, we simplified the equations into a 1D problem ($\sim e^{i\omega t+iqr}$) with scalar wavenumbers and speeds. For podosome waves in a 2D space ($\sim e^{i\omega t+i\vec{q}\cdot\vec{r}}$), the velocity-measured STICS can be considered as phase speed $v = \omega/|\vec{q}|$, where the direction follows the wave vector direction $\vec{q}/|\vec{q}|$. In other words, if we approximate our simulated wave dynamics of podosome height as a function $h(\omega t + \vec{q}\cdot\vec{r})$, the wave velocity directionality comes from the gradient of core height $\vec{\nabla}h$, while its magnitude can be estimated as $(dh/dt)/|\vec{\nabla}h|$.

Supplementary Note 6. STICS characterizes wave dynamics in the podosome cluster

Previous studies have applied spatiotemporal image correlation spectroscopy (STICS) to podosome systems in dendritic cells, and the propagation of fluorescence intensity for a variety of fluorescently labelled podosome components has been measured[13,26]. In practice, STICS measures the spatial correlations of an image within a region of interest (ROI) and then evaluates their persistence over sequential images separated by a time lag. The calculated spatial correlation function for a given time lag is the average of the spatial correlations between the separated frame pairs iterated over the time window of the STICS measurement. Velocity vectors can be mapped by measuring the translation of spatial correlation peaks as a function of the time lag (refer to Supplementary Methods in our previous work[26]).

In the podosome cluster of dendtric cells, we can consider that podosomes display wave-like dynamics in two distinct spatiotemporal scales: lateral displacement of individual podosomes and height waves throughout the entire cluster. For lateral displacement of individual podosomes, individual podosomes experience reorganization events due to actin polymerization and depolymerization processes. This leads to changes in their shape and to the apparent lateral displacement of the podosomes, as well as to splitting and merging events as the architecture of the podosomes is reorganized. Different from the lateral displacement of podosomes, the

coordination of podosome height oscillations leads to the emergence of F-actin intensity waves that propagate within podosome clusters. These dynamics can span significantly larger spatial scales than individual podosome dynamics and sometimes even the entire cell. In our work, we provide a theoretical model for the molecular mechanisms that explain the emergence of these height waves.We can consider the radial waves in a podosome cluster as an example (Supplementary Figure 10A), where we assumed an initial distribution where only the podosomes at the center have large heights. We take a closer look at the dynamics of individual podosomes at three instances of time (Supplementary Figures 10A-C):

T1: Podosome a has a larger height (above the steady-state height). Podosome a depolymerizes, leading to a decrease in its height, while podosomes b and c remain close to steady state (Supplementary Figure 10A).

T2: As podosome a depolymerizes, the newly released G-actin diffuse outward, altering the G-actin concentration near the podosome b and disrupting the previous balance. Podosome b starts growing and reaches its maximum height. The wavefront moves to the rim where podosome b is located (Supplementary Figures 10B-C)

T3: Podosome b begins to depolymerize, sending G-actin further outwards to activate the oscillation of podosome c. When the podosome c reaches its maximum height, the rim where podosome c is located becomes the wavefront (Supplementary Figure 10A).

As a result, although the podosomes (e.g., a, b, c) are not moving, the position with the largest podosome heights travels in a wave-like fashion. For example, the wave speed for time T2 is $v_c = (L_{w1} + L_{w2})/(T_3 - T_1)$, as shown in Supplementary Figure 10B. Mathematically, one can consider the podosome height waves $\delta h \sim e^{i\omega t + iqr}$ (with the linear dispersion $\omega \propto \mathcal{R}_e(q)$), and the phase speed is $v_c = \omega/\mathcal{R}_e(q)$.

Due to the correlation approach adopted by STICS as we mentioned before, we can take advantage of the temporal and spatial parameters to select which spatiotemporal regimes to preferentially pick-up. By doing so, we can increase our capacity to detect podosome height waves by selecting a big enough ROI and a small enough TOI, while minimizing the contribution of podosome lateral displacements, which would be better captured using a smaller ROI and an even smaller TOI. Leveraging this tuning capacity has been the rationale behind our decision to use STICS in this work and our previous publication[26]. To show the capability of STICS in

characterizing the wave height dynamic, we have selected representative regions of LifeAct-RFP transfected dendritic cells and made a visual summary, which clearly shows that the STICS vectors can guide the transition of high fluorescence intensity of podosomes (Supplementary Figure 8C). For example, in Example Flow 1, when the arrows are pointing towards left, the podosome 2 becomes brighter while the podosome 1 becomes darker; in Example Flow 2, when the STICS arrows are pointing towards the centre, the podosome 1 in the centre position appears. This indicates the F-actin flow or wave propagation follows the STICS arrow directions. Next, we also performed STICS on the simulated podosome dynamics based on our theoretical model (Eqs. 5-7) to show capability of STICS in characterizing wave dynamics. Our analysis shows that the STICS vectors (arrows in Supplementary Figure 9A and Supplementary Movie 5) can characterize the transition of the positions with maximal podosome heights despite the fast fusion and disassociation of podosomes (Supplementary Figures 9A-C). Interestingly, by plotting the histogram of velocity magnitudes measured by STICS (Supplementary Figure 9B), we found the magnitudes of flow velocities are around $0.1\ \mu m/min$ which is close to our theoretically-predicted wave speed $\frac{\lambda}{T} \sim \frac{3\ \mu m}{7\ min}$. Together, the STICS analysis on experiments (Supplementary Figure 8C) and simulations (Supplementary Figure 9) shows that measured velocity vectors can characterize the dynamics of waves qualitatively.

Supplementary References:

1 Bouissou, A. *et al.* Podosome Force Generation Machinery: A Local Balance between Protrusion at the Core and Traction at the Ring. *ACS Nano* **11**, 4028-4040, doi:10.1021/acsnano.7b00622 (2017).

2 Labernadie, A. *et al.* Protrusion force microscopy reveals oscillatory force generation and mechanosensing activity of human macrophage podosomes. *Nat. Commun.* **5**, 5343, doi:10.1038/ncomms6343 (2014).

3 Chan, C. E. & Odde, D. J. Traction dynamics of filopodia on compliant substrates. *Science* **322**, 1687-1691, doi:10.1126/science.1163595 (2008).

4 Gong, Z. *et al.* Recursive Feedback between Matrix Dissipation and Chemo-Mechanical Signaling Drives Oscillatory Growth of Cancer Cell Invadopodia. *Cell Rep.* **35**, 109047 (2021).

5 Johnston, A. B., Collins, A. & Goode, B. L. High-speed depolymerization at actin filament ends jointly catalysed by Twinfilin and Srv2/CAP. *Nat. Cell Biol.* **17**, 1504-1511 (2015).

6 Mogilner, A. & Oster, G. Force generation by actin polymerization ii: The elastic ratchet and tethered filaments. *Biophys. J.* **84**, 1591-1605, doi:10.1016/S0006-3495(03)74969-8 (2003).

7 Bangasser, B. L. & Odde, D. J. Master equation- based analysis of a motor- clutch model for cell traction force. *Cell. Mol. Bioeng.* **6**, 449-459, doi:10.1007/s12195-013-0296-5 (2013).

8 Shenoy, V. B., Wang, H. & Wang, X. A chemo-mechanical free-energy-based approach to model durotaxis and extracellular stiffness-dependent contraction and polarization of cells. *Interface Focus* **6**, 20150067, doi:10.1098/rsfs.2015.0067 (2016).
9 Mogilner, A. & Rubinstein, B. The physics of filopodial protrusion. *Biophys. J.* **89**, 782-795, doi:10.1529/biophysj.104.056515 (2005).
10 Lan, Y. & Papoian, G. A. The stochastic dynamics of filopodial growth. *Biophys. J.* **94**, 3839-3852, doi:10.1529/biophysj.107.123778 (2008).
11 He, B., Martin, A. & Wieschaus, E. Flow-dependent myosin recruitment during Drosophila cellularization requires zygotic dunk activity. *Development* **143**, 2417-2430, doi:10.1242/dev.131334 (2016).
12 Marchenko, O. O. *et al.* A minimal actomyosin-based model predicts the dynamics of filopodia on neuronal dendrites. *Mol. Biol. Cell* **28**, 1021-1033 (2017).
13 van den Dries, K. *et al.* Modular actin nano-architecture enables podosome protrusion and mechanosensing. *Nat. Commun.* **10**, 5171, doi:10.1038/s41467-019-13123-3 (2019).
14 Proag, A. *et al.* Working together: spatial synchrony in the force and actin dynamics of podosome first neighbors. *ACS nano* **9**, 3800-3813 (2015).
15 Mogilner, A. & Oster, G. Cell motility driven by actin polymerization. *Biophys. J.* **71**, 3030-3045, doi:10.1016/S0006-3495(96)79496-1 (1996).
16 Banerjee, D. S., Munjal, A., Lecuit, T. & Rao, M. Actomyosin pulsation and flows in an active elastomer with turnover and network remodeling. *Nat. Commun.* **8**, 1-13 (2017).
17 Dierkes, K., Sumi, A., Solon, J. & Salbreux, G. Spontaneous oscillations of elastic contractile materials with turnover. *Phys. Rev. Lett.* **113**, 148102, doi:10.1103/PhysRevLett.113.148102 (2014).
18 Labernadie, A., Thibault, C., Vieu, C., Maridonneau-Parini, I. & Charrière, G. M. Dynamics of podosome stiffness revealed by atomic force microscopy. *Proceedings of the National Academy of Sciences* **107**, 21016-21021 (2010).
19 Collin, O. *et al.* Spatiotemporal dynamics of actin-rich adhesion microdomains: influence of substrate flexibility. *J. Cell Sci.* **119**, 1914-1925, doi:10.1242/jcs.02838 (2006).
20 Jasnin, M. *et al.* Elasticity of podosome actin networks produces nanonewton protrusive forces. *Nat. Commun.* **13**, doi:10.1038/s41467-022-30652-6 (2022).
21 Gong, Z. *et al.* Matching material and cellular timescales maximizes cell spreading on viscoelastic substrates. *Proc. Natl. Acad. Sci. U. S. A.* **115**, E2686-E2695, doi:10.1073/pnas.1716620115 (2018).
22 Shi, Z., Graber, Z. T., Baumgart, T., Stone, H. A. & Cohen, A. E. Cell Membranes Resist Flow. *Cell* **175**, 1769-1779.e1713, doi:10.1016/j.cell.2018.09.054 (2018).
23 Liu, C., Beratan, D. N. & Zhang, P. Coarse-grained theory of biological charge transfer with spatially and temporally correlated noise. *J. Phys. Chem. B* **120**, 3624-3633 (2016).
24 Wang, S., Liu, F., Wang, W. & Yu, Y. Impact of spatially correlated noise on neuronal firing. *Physical Review E* **69**, 011909 (2004).
25 Lindner, B., Doiron, B. & Longtin, A. Theory of oscillatory firing induced by spatially correlated noise and delayed inhibitory feedback. *Physical Review E* **72**, 061919 (2005).
26 Meddens, M. B. *et al.* Actomyosin-dependent dynamic spatial patterns of cytoskeletal components drive mesoscale podosome organization. *Nat. Commun.* **7**, 13127, doi:10.1038/ncomms13127 (2016).
