# Supplementary material for: Chemo-mechanical diffusion waves explain collective dynamics of immune cell podosomes
Source: Nat Commun. 2023 May 22;14:2902. doi: 10.1038/s41467-023-38598-z (PMC10202956; doi:10.1038/s41467-023-38598-z)
Supplement: Supplementary file 3 — Description of additional supplementary files [file 41467_2023_38598_MOESM3_ESM.pdf]

Description of additional supplementary files

Supplementary Movie 1: Podosome cluster in a representative DC with LifeAct-RFP and vinculin-GFP transfected showing oscillations of individual podosomes and random waves in podosome cluster.

Supplementary Movie 2: Simulated podosome cluster with the discrete approach showing the propagation of radial and random waves.

Supplementary Movie 3: Simulated podosome cluster with the continuum approach showing the propagation of radial and random waves.

Supplementary Movie 4: Representative same DC before and after adding cytochalasin D.DCs are transfected with LifeAct-RFP using confocal microscopy with STICS analysis shown as vector maps.

Supplementary Movie 5: The STICS analysis for a simulated podosome cluster with the theoretical (continuum) approach.
