# Supplementary material for: Chemo-mechanical diffusion waves explain collective dynamics of immune cell podosomes
Source: Nat Commun. 2023 May 22;14:2902. doi: 10.1038/s41467-023-38598-z (PMC10202956; doi:10.1038/s41467-023-38598-z)
Supplement: Supplementary file 9 — Reporting Summary [file 41467_2023_38598_MOESM9_ESM.pdf]

nature portfolio

Corresponding author(s): Vivek B. Shenoy

Last updated by author(s): Apr 19, 2023

Reporting Summary

Nature Portfolio wishes to improve the reproducibility of the work that we publish. This form provides structure for consistency and transparency in reporting. For further information on Nature Portfolio policies, see our Editorial Policies and the Editorial Policy Checklist.

Statistics

For all statistical analyses, confirm that the following items are present in the figure legend, table legend, main text, or Methods section.

n/a	Confirmed	
☐	☒	The exact sample size (*n*) for each experimental group/condition, given as a discrete number and unit of measurement
☐	☒	A statement on whether measurements were taken from distinct samples or whether the same sample was measured repeatedly
☐	☒	The statistical test(s) used AND whether they are one- or two-sided<br>*Only common tests should be described solely by name; describe more complex techniques in the Methods section.*
☒	☐	A description of all covariates tested
☒	☐	A description of any assumptions or corrections, such as tests of normality and adjustment for multiple comparisons
☐	☒	A full description of the statistical parameters including central tendency (e.g. means) or other basic estimates (e.g. regression coefficient) AND variation (e.g. standard deviation) or associated estimates of uncertainty (e.g. confidence intervals)
☐	☒	For null hypothesis testing, the test statistic (e.g. *F*, *t*, *r*) with confidence intervals, effect sizes, degrees of freedom and *P* value noted<br>*Give P values as exact values whenever suitable.*
☒	☐	For Bayesian analysis, information on the choice of priors and Markov chain Monte Carlo settings
☒	☐	For hierarchical and complex designs, identification of the appropriate level for tests and full reporting of outcomes
☐	☒	Estimates of effect sizes (e.g. Cohen's *d*, Pearson's *r*), indicating how they were calculated

Our web collection on statistics for biologists contains articles on many of the points above.

Software and code

Policy information about availability of computer code

Data collection	Matlab R2021a and COMSOL 5.5 are used for simulating podosome dynamics; MATLAB and COMSOL files used in this work are openly available on Github (https://github.com/gongze/Podosome_wave).
Data analysis	Fiji 2.9.0 and Matlab R2021a are used to analyze the experimental data in this study.

For manuscripts utilizing custom algorithms or software that are central to the research but not yet described in published literature, software must be made available to editors and reviewers. We strongly encourage code deposition in a community repository (e.g. GitHub). See the Nature Portfolio guidelines for submitting code & software for further information.

Data

Policy information about availability of data

All manuscripts must include a data availability statement. This statement should provide the following information, where applicable:

- Accession codes, unique identifiers, or web links for publicly available datasets
- A description of any restrictions on data availability
- For clinical datasets or third party data, please ensure that the statement adheres to our policy

All the data supporting the findings described in this study are archived and publicly available via All the data supporting the findings described in this study are archived and publicly available via 4TU.Research Data (https://doi.org/10.4121/183632e9-29e9-4a20-ac38-296bda95fc6d).

Research involving human participants, their data, or biological material

Policy information about studies with human participants or human data. See also policy information about sex, gender (identity/presentation), and sexual orientation and race, ethnicity and racism.

Reporting on sex and gender	N/A
Reporting on race, ethnicity, or other socially relevant groupings	N/A
Population characteristics	N/A
Recruitment	N/A
Ethics oversight	N/A

Note that full information on the approval of the study protocol must also be provided in the manuscript.

Field-specific reporting

Please select the one below that is the best fit for your research. If you are not sure, read the appropriate sections before making your selection.

☒ Life sciences ☐ Behavioural & social sciences ☐ Ecological, evolutionary & environmental sciences

For a reference copy of the document with all sections, see nature.com/documents/nr-reporting-summary-flat.pdf

Life sciences study design

All studies must disclose on these points even when the disclosure is negative.

Sample size	No statistical methods were used to predetermine sample size and choices were largely based on common practice in the field. Specifically, for our experiments, we used at least 5 different cells from 2 independent experiments for the single cell live cell imaging in all of the conditions. This number is sufficient since we extract quantitative information from all of the podosomes for each of the frames by using STICS. Furthermore, we have consistently noted that although the cells themselves are heterogeneous in shape and size, quantitative parameters describing podosome behaviour (oscillation speed, wave speed etc) are remarkably similar among cells resulting in little cell to cell variation.
Data exclusions	No exclusion.
Replication	All the measurements were done at least three independent experiments to verify the reproducibility of the experimental findings, and the results were consistent.
Randomization	Results were obtained from single cell live imaging experiments of dendritic cells transfected with fluorescent fusion constructs. For all conditions (control or treated), cells positive for the fluorescent fusion protein were selected and imaged. For this, dendritic cells were chosen in a random position within the well and the only requirement for selection was that the cell formed a podosome cluster that is representative for the given condition. Cells that did not have any podosomes were not imaged.
Blinding	The investigation were blinded to group allocation during data collection and analysis for drug/stiffness perturbation results.

Reporting for specific materials, systems and methods

We require information from authors about some types of materials, experimental systems and methods used in many studies. Here, indicate whether each material, system or method listed is relevant to your study. If you are not sure if a list item applies to your research, read the appropriate section before selecting a response.

Materials & experimental systems

n/a	Involved in the study
☒	☐ Antibodies
☒	☐ Eukaryotic cell lines
☒	☐ Palaeontology and archaeology
☒	☐ Animals and other organisms
☒	☐ Clinical data
☒	☐ Dual use research of concern
☒	☐ Plants

Methods

n/a	Involved in the study
☒	☐ ChIP-seq
☒	☐ Flow cytometry
☒	☐ MRI-based neuroimaging
